# Supplementary material for: Modulating the Graphitic Domains and Pore Structure of Corncob-Derived Hard Carbons by Pyrolysis to Improve Sodium Storage
Source: Molecules. 2023 Apr 20;28(8):3595. doi: 10.3390/molecules28083595 (PMC10143560; doi:10.3390/molecules28083595)
Supplement: Supplementary file 1 [file molecules-28-03595-s001.zip › molecules-2285963-supplementary.pdf]

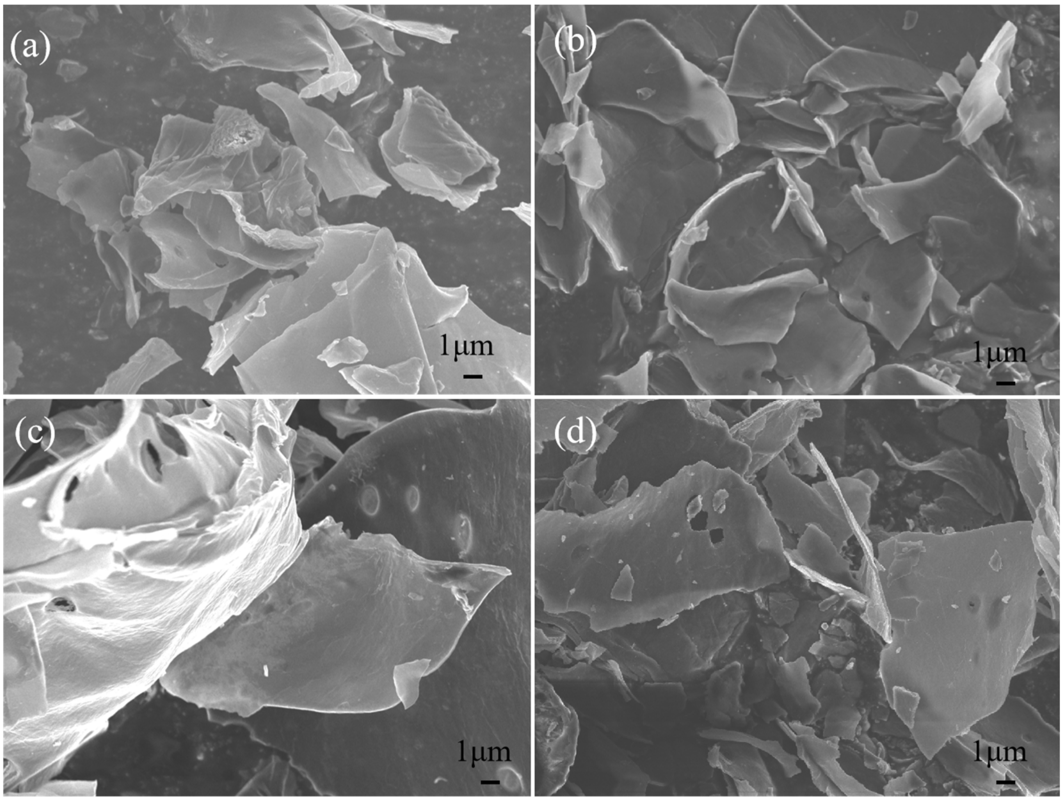

Figure S1. SEM images of CC-1000(a), CC-1200(b), CC-1400(c), CC-1600(d).

Table S1. The percent of C, O atoms and functionalities calculated by XPS.

	**C atoms(%)**	**O atoms(%)**	**C=C(%)**	**C-C(%)**	**C-O(%)**	**C=O(%)**
CC-1000	92.37	7.20	25.80	44.56	13.05	8.96
CC-1200	93.94	5.96	27.61	44.04	13.94	8.85
CC-1400	95.45	2.87	29.91	42.32	15.98	7.24
CC-1600	94.89	5.02	26.65	48.30	15.57	4.37
